# Management of clinical relevant drug-drug interactions with antipsychotics in nursing homes

**DOI:** 10.1080/07853890.2021.1896843

**Published:** 2021-09-28

**Authors:** Catarina Bernardo, João Pedro Aguiar, Filipa Alves da Costa

**Affiliations:** a Centro de Investigação Interdisciplinar Egas Moniz (CiiEM), Egas Moniz Cooperativa de Ensino Superior, Caparica, Portugal; b The Research Institute for Medicines (iMED.ULisboa), Faculdade de Farmácia, Universidade de Lisboa, Lisboa, Portugal

## Abstract

**Introduction:**

Antipsychotics (APs) have been linked to several clinically relevant drug-drug interactions (leading, e.g. to QT-prolongation, torsades de pointes) but little is known about their management in nursing homes residents. Our aim was to characterise clinically relevant drug-drug interactions involving APs and to assess their prevalence in nursing homes residents.

**Materials and methods:**

We conducted a cross-sectional study in two nursing homes in Portugal. Patients were included if they were older ≥65 and prescribed at least one AP (registered in medical charts). Interactions were identified using Drug Interaction Chequer (Medscape [1]) and supplemented with the summary of product characteristics (SmPC) when deemed necessary. Clinical relevant interactions were defined as serious or contraindicated interactions according to Medscape tool. Data were analysed using univariate statistics (Microsoft Excel 2016).

**Results:**

A total of 59 patients (83.7 ± 7.1 years) were analysed, 79.7% (*n* = 47) females. A mean of 1.6 ± 0.7 antipsychotics were prescribed/patient, mostly on a regular basis (61.0%; *n* = 36). The majority were exclusively prescribed atypical APs (59.3%; *n* = 35), 28.8% (*n* = 17) simultaneously atypical and typical APs and 11.9% (*n* = 7) prescribed only typical APs. Nearly 90% (*n* = 52; 88.1%) of patients reported interactions with APs, (mean = 2.9 ± 2.3/patient). A total of 169 interactions with APs were found, mainly with antidepressants (27.2%; *n* = 46), benzodiazepines (22.4%; *n* = 38), and other antipsychotics (10.6%; *n* = 18). The majority of AP-drug interactions (83.4%; *n* = 141) were classified as “to be closely monitored”, mostly due to the sedation effect (56.7%; *n* = 80). Quetiapine was the AP most often involved in interactions (45.6%; *n* = 77), where 9.1% (*n* = 7) were considered “serious”. Haloperidol also registered 37 interactions (21.9%), four of which resulted in increased QTc interval (10.8%).

**Discussion and conclusions:**

Data showed that one third of patients had clinically relevant AP-drug interactions. Future work will include the development of a user-friendly booklet on clinical relevant drug-drug interactions in elderly patients using APs, expected to contribute to enhanced patient safety and a more rational use of medicines.

## References

[CIT0001] Medscape. Drug interactions checker [Internet]. 2019. Available from: https://reference.medscape.com/drug-interactionchecker.

